# Computational Modeling of Glucose Uptake in the Enterocyte

**DOI:** 10.3389/fphys.2019.00380

**Published:** 2019-04-12

**Authors:** Nima Afshar, Soroush Safaei, David P. Nickerson, Peter J. Hunter, Vinod Suresh

**Affiliations:** ^1^Auckland Bioengineering Institute, University of Auckland, Auckland, New Zealand; ^2^Department of Engineering Science, University of Auckland, Auckland, New Zealand

**Keywords:** computational modeling, glucose uptake, SGLT1, GLUT2, CellML, OpenCOR

## Abstract

Absorption of glucose across the epithelial cells of the small intestine is a key process in human nutrition and initiates signaling cascades that regulate metabolic homeostasis. Validated and predictive mathematical models of glucose transport in intestinal epithelial cells are essential for interpreting experimental data, generating hypotheses, and understanding the contributions of and interactions between transport pathways. Here we report on the development of such a model that, in contrast to existing models, incorporates mechanistic descriptions of all relevant transport proteins and is implemented in the CellML framework. The model is validated against experimental and simulation data from the literature. It is then used to elucidate the relative contributions of the sodium-glucose cotransporter (SGLT1) and the glucose transporter type 2 (GLUT2) proteins in published measurements of glucose absorption from human intestinal epithelial cell lines. The model predicts that the contribution of SGLT1 dominates at low extracellular glucose concentrations (<20 mM) and short exposure times (<60 s) while the GLUT2 contribution is more significant at high glucose concentrations and long durations. Implementation in CellML permitted a modular structure in which the model was composed by reusing existing models of the individual transporters. The final structure also permits transparent changes of the model components and parameter values in order to facilitate model reuse, extension, and customization (for example, to simplify, or add complexity to specific transporter/pathway models, or reuse the model as a component of a larger framework) and carry out parameter sensitivity studies.

## 1. Introduction

Almost all of the nutrients, electrolytes, and water from food are absorbed into blood capillaries through the mucosa of the small intestine. Most absorption processes in the small intestine are driven by an electrochemical gradient of ions across the boundary of epithelial cells (enterolyses) lining the lumen. Transporter proteins embedded in the apical membrane carry ions and nutrients into the enterocyte. Other transporters in the basolateral membrane then extrude the ions into the interstitial space from where they enter capillary blood by diffusion. Carbohydrates are the main source of energy in the body. They break down to monosaccharides like glucose, which is the most important carbohydrate fuel in the cell. Therefore the uptake and transport of glucose through the small intestine epithelial cells is a vital aspect of human nutrition. Subsequent transport and metabolism of the absorbed species triggers responses such as hormone release, appetite regulation and growth via complex physiological feedback pathways. A mechanistic understanding of these pathways and how they are disrupted in disease is lacking, partly due to the difficulties of making experimental measurements in the luminal and capillary compartments. A validated computational model of the absorption pathways can overcome these difficulties by providing quantitative predictions of concentrations and transport rates in the lumen and cell compartments (Hunter and Borg, 2003; Ingalls, 2012).

Many studies in the past few decades have focussed on mathematical modeling of the glucose-insulin control system in order to study how metabolism and the regulatory system are disrupted in diseases like diabetes (reviewed in Palumbo et al., 2013). At the cellular level, models of glucose uptake and transport in the kidneys (Weinstein, 2015), glucose homeostasis in the liver (König et al., 2012), and glucose sensing (Riz and Pedersen, 2015) have been developed. In contrast, mathematical modeling of glucose uptake by the enterolyses lining the small intestinal mucosa has attracted little attention. The first model of glucose transport in the enterocyte was developed by Thorsen et al. (2014). The model focussed on the regulation of Na,K-ATPase in enterolyses during glucose absorption. It considered SGLT1 as the sole pathway for glucose entry into the cell at the apical membrane and studied how the intracellular Na+ concentration can be maintained in the face of SGLT1-associated Na+ influx. One limitation of the model is the absence of a GLUT2 pathway for glucose entry at the apical membrane. The role of apical GLUT2 is still a matter of controversy with some studies indicating its presence and importance for glucose uptake (Kellett and Brot-Laroche, 2005; Zheng et al., 2012) while others have suggested SGLT1 as the dominant or sole pathway (Gorboulev et al., 2012; Röder et al., 2014). Differences in experimental conditions and data interpretation are partly the reason for lack of consensus (Kellett, 2012; Koepsell and Gorboulev, 2012). In this work, we developed a mathematical model that includes apical GLUT2 and parameterized it against published experimental data. We then used the model to examine the relative contributions of SGLT1 and GLUT2 in published cell culture data on glucose uptake (Zheng et al., 2012). Finally we assessed the impact of increased glucose transporter expression on uptake rates in diabetes.

The Thorsen model incorporated a mixture of mechanistic transporter models (e.g., SGLT1, basolateral GLUT2), empirical flux expressions (e.g., NaK-ATPase, an effective Na-Cl co-transporter), and diffusive membrane fluxes for Na+, K+, and Cl. We modified this framework to explicitly incorporate mechanistic models of all relevant transporters. In particular, we replaced the Na-Cl co-transporter in the original model with individual models for the anion exchanger 1 (AE1) and Na+ /H+ exchanger (NHE3) proteins at the apical membrane and incorporated ENaC and CFTR channels for apical Na+ and Cl− transport. This makes it possible to use the model to study scenarios where the expression and/or function of these transport proteins is altered, for example in gene knockout/mutation studies or the use of channel inhibitors and agonists.

The model is implemented in the open source, extensible markup language (XML)-based CellML modeling environment used to represent mathematical models of biology based on ordinary differential and algebraic equations (Cuellar et al., 2003). We adopted a modular, compositional approach to model construction by reusing CellML models of individual transport proteins encoded in an online, curated repository [Physiome Model Repository (PMR, models.physiomeproject.org)] to facilitate the sharing of models (Yu et al., 2011). The complete model, including parameter values, simulation software and simulation conditions, can be downloaded from PMR with the following link: https://models.physiomeproject.org/workspace/572.

## 2. Methods

### 2.1. Model Construction

**Figure 1:**
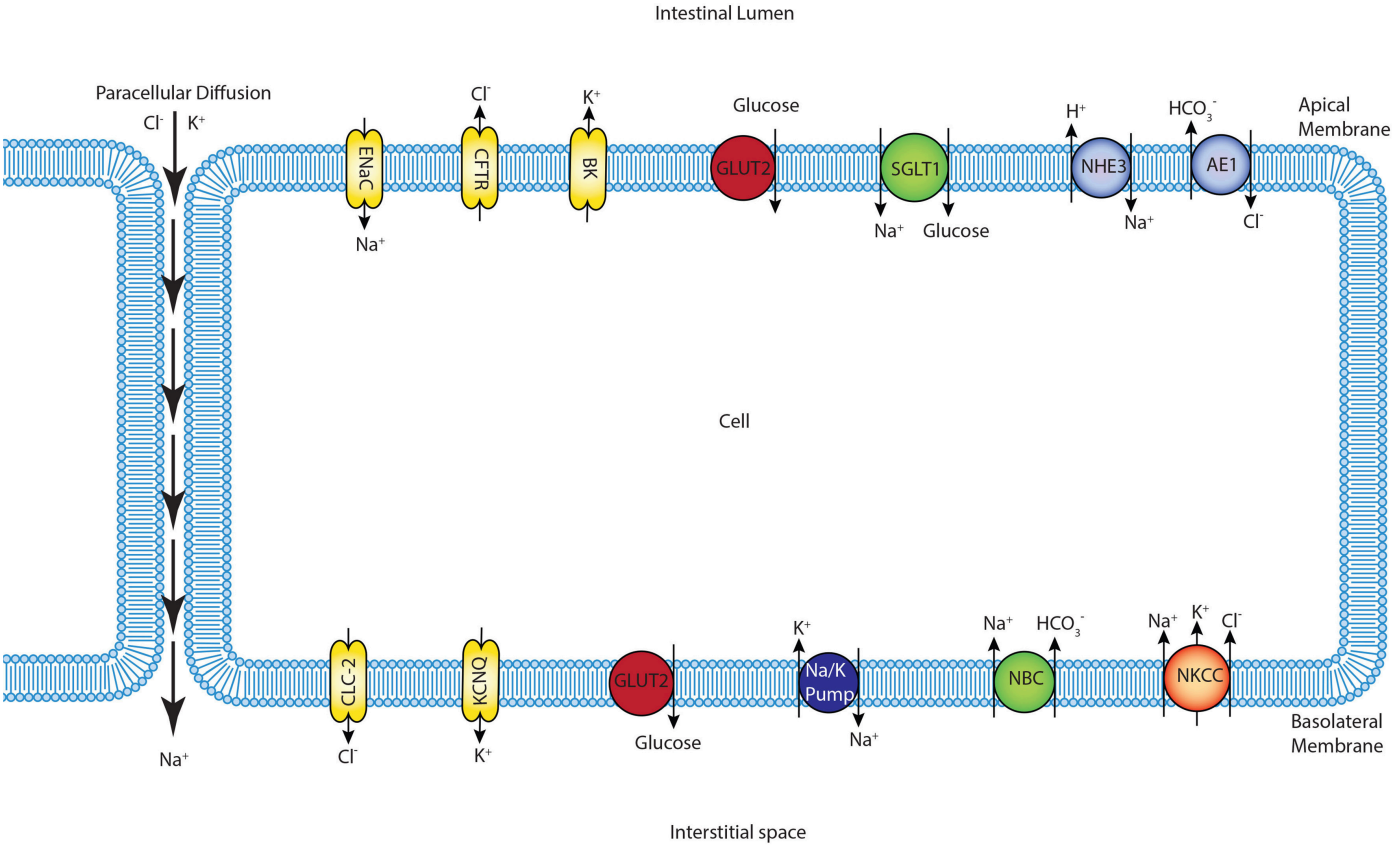
Schematic of enterocyte showing the relevant transporters in the apical and basolateral membrane along with the apical (lumen) and basolateral (interstitium) extracellular domains.

We constructed a mathematical model of the epithelial cell of a small intestine (enterocyte) that incorporates the relevant transport proteins identified in the literature (Barrett and Keely, 2015) and diffusion pathways (Figure 1). The membrane localization and function of these transporters and the source of the original mathematical models are listed in Table 1. The apical (luminal) and basolateral (interstitial) surface of the cell are in contact with distinct extracellular compartments. Transport of substances occurs across the membranes as well as directly between the extracellular compartments across the paracellular junctions. The variables to be solved in the model are chemical species (Na^+^, K^+^, H^+^, Cl^−^, ${\text{HCO}}_{3}^{-}$, glucose) concentrations in each compartment and the two membrane potentials. Flux balance and electric charge conservation laws yield the governing equations of the model. Water transport is not included and hence we limit ourselves to modeling iso-osmotic transport. Model equations are provided in the Supplementary Material.

**Table 1:** List of transporters used in the model along with their locations and roles.

**Transporter**	**Location**	**Role**	**Chemical Species**	**Source of the mathematical model**
SGLT1	Apical	Cotransporter	1 Glucose, 2 Na^+^	Parent et al., 1992
NaK ATPase	Basolateral	Exchange Pump	3Na^+^, 2K^+^	Thorsen et al., 2014
GLUT2	Apical and Basolateral	Uniporter Protein	Glucose	Pradhan et al., 2013
NHE3	Apical	Antiporter	1 Na^+^, 1 H^+^	Weinstein, 1995
AE1	Apical	Antiporter	1 Cl^−^, 1 ${\text{HCO}}_{3}^{-}$	Weinstein, 2000
BK	Apical	Channel	_*K*_+	Fong et al., 2016
CFTR	Apical	Channel	Cl^−^	Fong et al., 2016
CLC-2	Basolateral	Channel	Cl^−^	Fong et al., 2016
ENaC	Apical	Channel	Na^+^	Fong et al., 2016
IK	Basolateral	Channel	K^+^	Fong et al., 2016
NBC	Basolateral	Cotransporter	1 Na^+^, 3 ${\text{HCO}}_{3}^{-}$	Østby et al., 2009
NKCC1	Basolateral	Cotransporter	1 Na^+^, 1 K^+^, 2 Cl^−^	Palk et al., 2010

**Figure 2:**
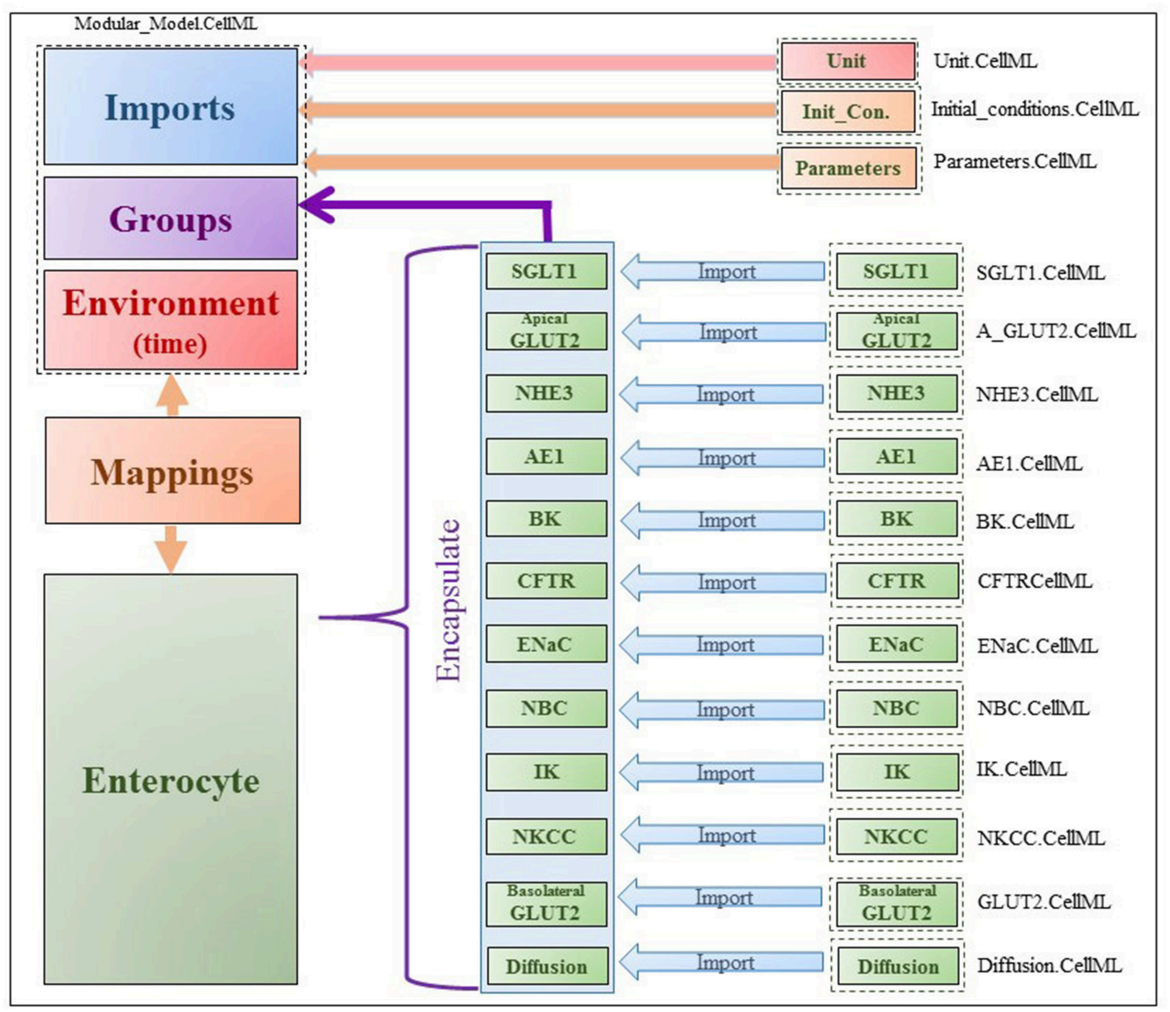
Figure shows the modularity of CellML model. Encapsulation hierarchy (purple), the CellML model imports (blue) and the other key parts (units, parameters, components and mappings) of the top level CellML model.

The model was implemented in the open-source, modular CellML framework. CellML is an XML based language commonly used to encode and simulate mathematical models based on algebraic and ordinary differential equations. Encoded models are available in an online, curated repository [Physiome Model Repository-PMR(models.physiomeproject.org)] (Yu et al., 2011). Reuse of models and components within models is possible through the use of the import element that enables encapsulation of other CellML files within a CellML model and facilitates a modular, compositional approach to the construction of complex models. The application of this approach in the enterocyte model is shown in Figure 2. Existing models of the individual transporters were imported into the top level model file (modular_model.cellml). Units and parameters for all components as well as initial conditions for specific simulations were specified in separate .cellml files and also imported into the top level file. The models were encapsulated as a group into the enterocyte component in which the overall balance equations for the chemical species and electric currents were coded. The mappings element links variables that are common between the different components, e.g., glucose concentrations in GLUT2.cellml, SGLT1.cellml and enterocyte. The environment component comprises independent variables that are common to all components, which in this case, is solely time.

The model was coded and simulated in OpenCOR (Version 0.5) (Garny and Hunter, 2015). The CVODES solver was used with the BDF integration method and Newton iterations. All of the models including their parameters can be downloaded from PMR with the following link: https://models.physiomeproject.org/workspace/572.

### 2.2. Comparison With Experiments

The model was validated against published experimental measurements of glucose uptake in the human enterocyte-like cell lines Caco-2 and IEC6 Zheng et al. (2012). In the experiments, the cells were cultured on impermeable surfaces for 10–15 days in high glucose (25 mM) medium. To measure glucose uptake, varying concentrations (0.5–50 mM) of glucose were introduced into the apical chamber in a buffer solution with a baseline composition 130 mM NaCl, 4 mM KH_2_PO_4_, 1 mM CaCl_2_. The osmolarity of the buffer was maintained during the measurements by modulating the NaCl content such that if the glucose concentration was *x* mM, NaCl concentration was 130−*x*/2 mM. After exposure to the glucose stimulus for different durations (30–600 s), cells were lysed and intracellular glucose and protein concentrations were measured. Since the measurements were reported in nanomole glucose per milligram (mg) protein, the data were converted to concentration units (millimole per liter, mM) by doing the unit conversion from nanomole/*m*^3^ to mM and also multiplying by the cellular protein concentration (mg protein per ml cell volume). The conversion factor *a* (protein density) was used as a fitting parameter in a non-linear Generalized Reduced Gradient optimization to match model outputs to the data. The optimization was done using the Microsoft Excel Solver (Microsoft Office 2013) by minimizing the least square error between model predicted and measured intracellular glucose concentration.

In the simulations, the apical compartment was treated as an infinite bath of constant composition based on the experimental conditions. Since the cells were cultured on an impermeable substrate, the volume of the basolateral compartment (*V*_*b*_) was not measured. In the simulations, *V*_*b*_ was fixed at different multiples (*m* = 0.1, 1, 10) of the cell volume (*V*_*c*_) and also as an infinite bath to generate a range of predictions. This allowed us to account for the uncertainty in the actual volume of the basolateral compartment. For finite values of *V*_*b*_, the composition of the basolateral compartment cannot be regarded as constant and was instead determined by the flux of glucose/ions across the basolateral membrane. Since the experiments were conducted under iso-osmotic conditions, there is no water transfer between the compartments and hence *V*_*b*_ and *V*_*c*_ were held fixed for the duration of each simulation.

## 3. Results

### 3.1. Steady State and Dynamic Responses

The model was first checked for physiological consistency by determining intracellular concentrations and membrane potentials in the absence and presence of a glucose stimulus. Steady state values of the model variables were computed with no glucose in the extracellular compartments. In these simulations, the composition of the apical and basolateral compartments were identical and held constant (140 mM Na^+^, 5.4 mM K^+^, 103 mM Cl^−^). Results were consistent with reported values (Table 2).

**Table 2:** Reported values for intracellular ions concentration from simulated model and literature.

**Ion**	**Model result**	**Reported** **value**	**Reference**
Na^+^ (mM)	61	45–65	Nellans and Schultz, 1976; Okada et al., 1976
K^+^ (mM)	127	120–40	Okada et al., 1976; Vogalis, 2000
Cl^−^ (mM)	69	50–70	Frizzell et al., 1973; Nellans et al., 1973; Okada et al., 1976
Apical (lumen-cell) membrane potential (mV)	−30	−36 ± 0.5	Rose and Schultz, 1971
Basolateral (interstitium-cell) membrane potential (mV)	−36	−40.5 ± 0.8	Rose and Schultz, 1971
pH	7.16	7.2	Shimada and Hoshi, 1987

**Figure 3:**
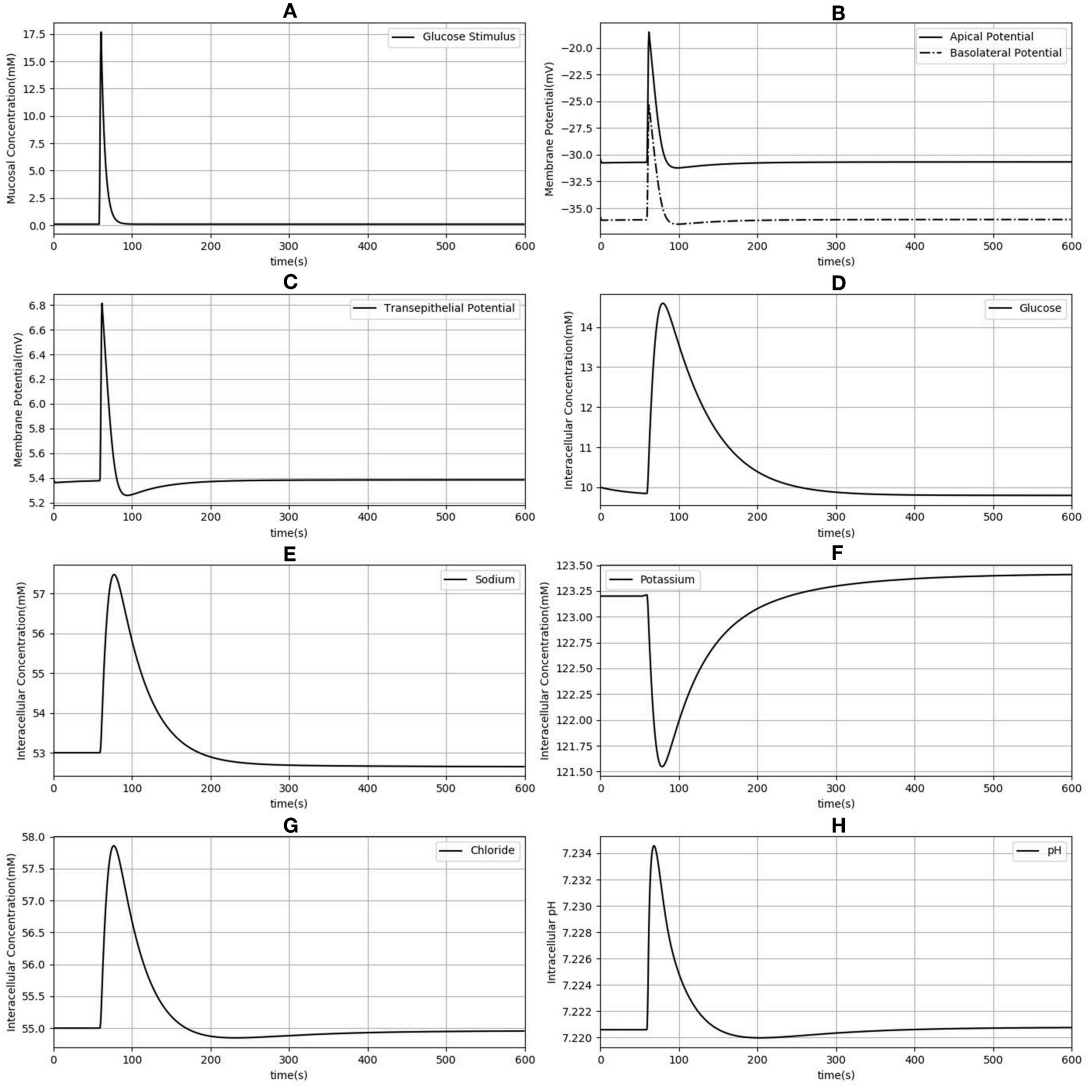
Dynamic response of the model to an extracellular glucose stimulus. The stimulus consists of a step increase followed by an exponential decay **(A)**. Apical and basolateral membrane potentials **(B)**, transepithelial potential **(C)**, and intracellular concentrations of glucose **(D)**, sodium **(E)**, potassium **(F)**, chloride **(G)**, and pH **(H)** are shown.

Next, the dynamic response to an apical glucose stimulus was determined. The model was initialized in the steady state described in Table 2 and a time dependent, extracellular glucose stimulus previously used in the literature (Thorsen et al., 2014) was applied at *t* = 60*s* (Figure 3A). Other extracellular variables were maintained at the same values used for the previous set of simulations. The stimulus causes a depolarization of both membranes (Figure 3B). Membrane potentials recover rapidly to baseline after around 100 s and mirror the time course of the stimulus. Transient changes in the transepithelial potential difference (≈ 1.4 mV increase) are of the same direction and comparable magnitude to values reported in the literature (1.9 ± 0.1 mV ;Rose and Schultz, 1971) while changes in the apical potential (≈ 12 mV increase) are higher than values reported in the same study (6 ± 0.5 mV) (Figures 3B,C). Intracellular ion concentrations and pH all exhibit a slower transient response than the membrane potentials that lasts for ≈200–300 s.

### 3.2. Comparison With the Thorsen et al. (2014) Model

**Figure 4:**
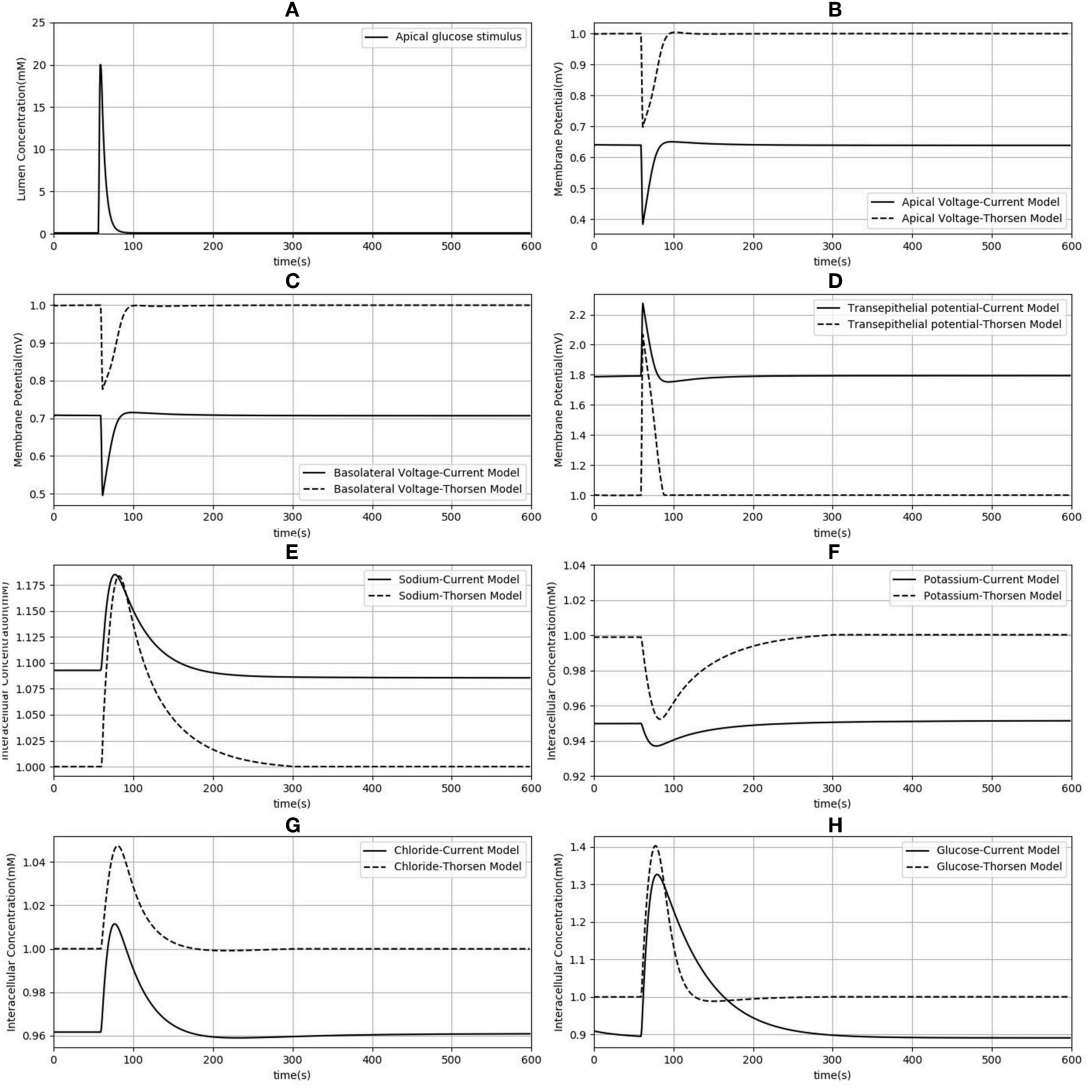
Comparison of model responses against the model of Thorsen et al. (2014). Each variable has been normalized against the corresponding steady value from the Thorsen model. **(A)** Apical glucose stimulus. **(B,C)** Apical and basolateral membrane potential respectively. **(D)** Transepithelial potential. **(E–H)** Sodium, potassium, chloride and glucose intracellular concentration.

Since our model is similar to that developed by Thorsen et al. (2014), we compared the responses of both models when the same parameters (Table 3), initial conditions and glucose stimulus were used. Model outputs were normalized against the steady state values of the Thorsen model and are shown in Figure 4. A few observations may be made: in the absence of a glucose stimulus, steady state values of the membrane potentials in our model are around 30% lower while the transepithelial potential is around 80% higher. Steady state values for concentration of chloride, potassium, and glucose are 5−10% lower than the values in the previous model whereas for sodium it is about 10% higher. In response to a glucose stimulus, our model has a larger change in membrane potentials and intracellular glucose, but smaller changes in sodium and potassium. Chloride responses are of almost the same magnitude in both models. The duration of the transients are similar in both models, except for glucose where our model has a similar rise time, but a slower decay (around 2 times slower).

**Table 3:** Parameter values used in the simulations.

**Parameter**	**Value in our model** **(Figures 3, 4)**	**Value in our model** **(Other figures)**	**Unit**
nSGLT1	18 × 10^7^	4 × 10^7^	–
nA_GLUT2_	0	42 × 10^7^	–
nB_GLUT2_	14 × 10^6^	14 × 10^7^	–
V_cell_	6 × 10^−16^	2 × 10^−15^	m^3^
Capacitance	1 × 10^−5^	1 × 10^−5^	μF

### 3.3. Comparison Against Cell Culture Data

Finally, model predictions were compared against measurements carried out in cell culture studies (Zheng et al., 2012). The experiments used Caco-2 and IEC6 cell lines. While Caco-2 expresses both SGLT1 and GLUT2, IEC6 cells do not express GLUT2. We therefore turned off the expression of GLUT2 in the apical membranes to simulate these cells.

**Figure 5:**
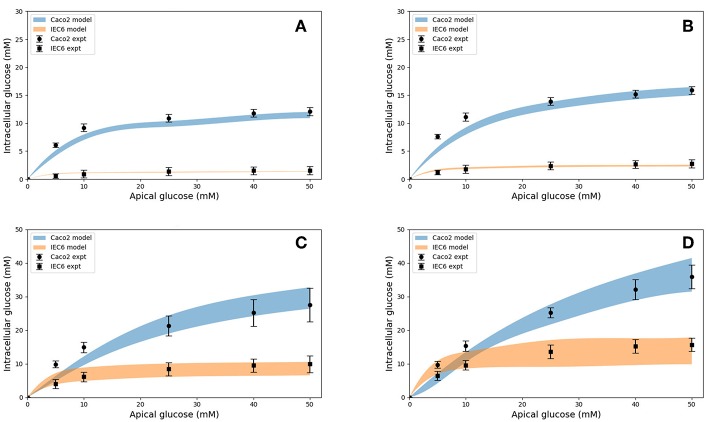
Intracellular glucose concentrations for a range of extracellular glucose concentrations in Caco2 and IEC6 cells and exposure times (**A**: 30 s, **B**: 60 s, **C**: 300 s, **D**: 600 s). Experimental data points and error bars were digitally extracted from Zheng et al. (2012). Strips for the model predictions represent the range of values generated by setting *V*_*b*_ = *mV*_*c*_, *m* = 0.1, 1, 10, 100, ∞.

Model predictions of the intracellular glucose concentrations are in good agreement with the measurements over the entire range of time points and apical glucose concentrations for both cell lines (Figure 5). As shown in Figures 5A,B at 30 and 60 s of exposure, glucose concentrations in both cell lines have a tendency to level off at higher concentration of glucose in the apical compartment. IEC6 still has the same behavior for longer exposure times (300 and 600 s) whereas concentrations in Caco2 do not saturate with increasing glucose concentration in the apical compartment (Figures 5C,D). The protein density parameter *a* are quite close to each other for Caco-2 cell line and varies for IEC-6 cell line to fit the four exposure durations (Table 4).

**Table 4:** Best fit values of the protein density (*a*) used to generate the simulated curves in Figure 5 for different exposure times and both cell lines.

**Exposure duration** **(seconds)**	***a* (g protein/ml)** **Caco2**	***a* (g protein/ml)** **IEC6**
30	0.021	0.009
60	0.026	0.013
300	0.032	0.035
600	0.03	0.047

Together, these results indicate that the model is able to reproduce a range of independent experimental observations. Next we present applications of the validated model to address questions about glucose uptake pathways in health and disease.

### 3.4. Role of Apical GLUT2 in Glucose Uptake

**Figure 6:**
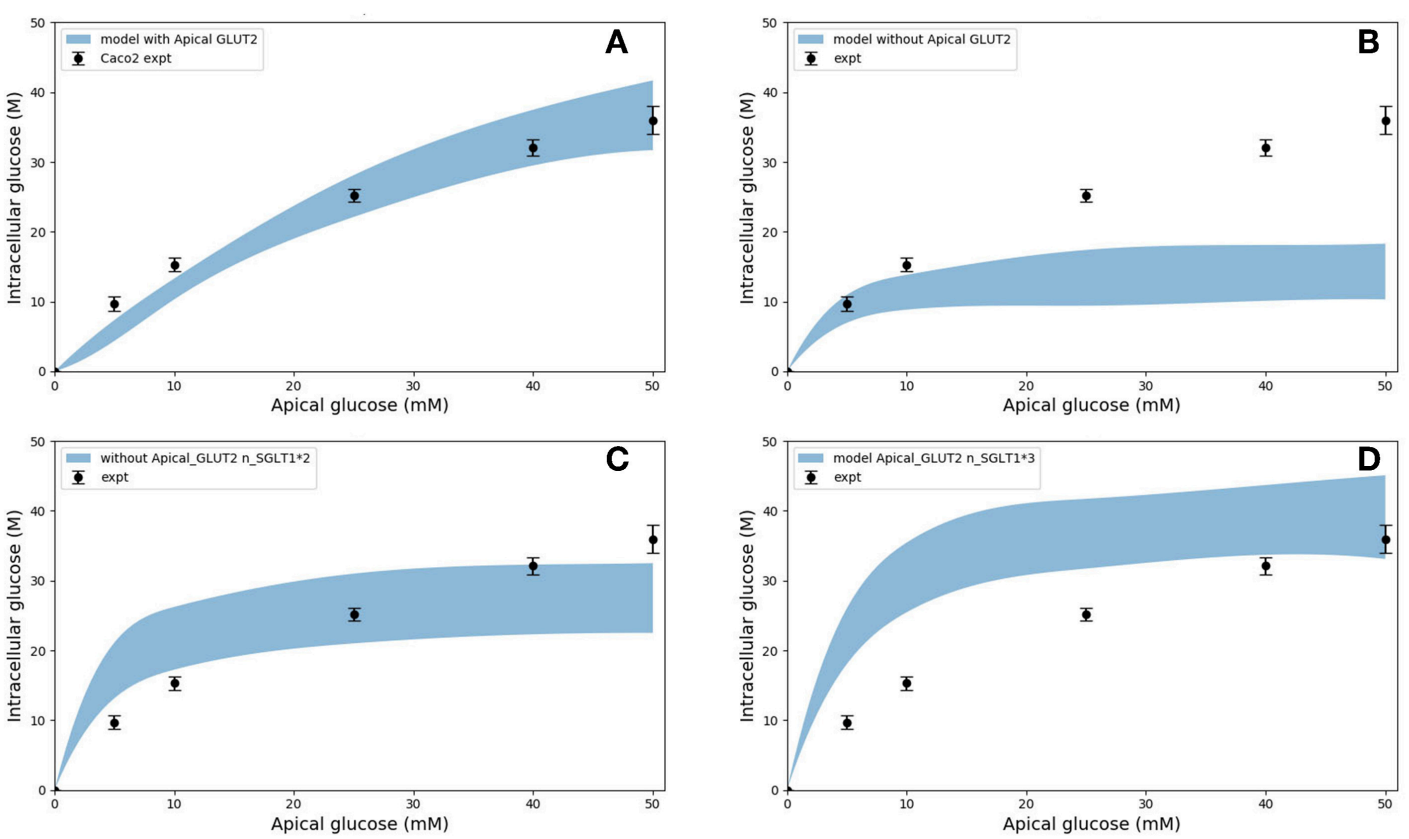
Intracellular glucose concentration vs. extracellular glucose concentration in Caco2 in the presence/absence of Apical GLUT2 with different number of SGLT1 transporter **(A)** Output of model with apical GLUT2 **(B)** Model does not have apical GLUT2 **(C)** model does not have apical GLUT2 and the number of SGLT1 is doubled **(D)** model does not have apical GLUT2 and the number of SGLT1 is 3-fold higher. Experimental data points and error bars were digitally extracted from Zheng et al. (2012). Strips for the model predictions represent the range of values generated by setting *V*_*b*_ = *mV*_*c*_, *m* = 0.1, 1, 10, 100, ∞.

In the original study of Zheng et al., the experimental data in Figures 5A–D were interpreted as indicating the presence of GLUT2-mediated uptake at the apical membrane (Zheng et al., 2012). We investigated if an alternative explanation was possible whereby SGLT1 expression levels in the model could be tuned to reproduce the same trends in intracellular glucose concentration. In Figures 6A–D, the data for Caco-2 cells at the 600 s time point are compared to the model with varying levels of apical GLUT2 and SGLT1. The baseline model with normal expression of SGLT1 and apical GLUT2 provides a good fit to the data over the full range of apical glucose concentrations (Figure 6A). When apical GLUT2 is turned off with no changes in SGLT1 expression (Figure 6B), model predictions of intracellular glucose are low compared to the data for apical glucose concentrations higher than 10 mM. In addition, model predictions saturate after around 20 mM of apical glucose while the data shows an increasing trend. A higher expression of SGLT1 was also examined and can provide a better match to the data in the absence of apical GLUT2. With no apical GLUT2 and 2-fold levels of baseline SGLT1 (Figure 6C) the model overpredicts the data at low apical glucose concentrations (<10 mM) and underpredicts the data at apical glucose concentrations >40 mM. When SGLT1 levels are increased to 3 times the baseline value, the model overpredicts the data over the whole range, except at a apical glucose of 50 mM (Figure 6D).

**Figure 7:**
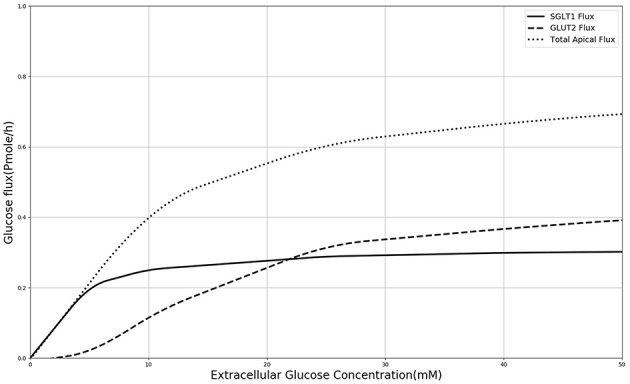
Glucose Flux through SGLT1 and GLUT2 in 600 s of simulation along with total apical flux of glucose.

In order to explain these results, the contribution of SGLT1 and GLUT2 to the apical glucose flux is shown in Figure 7 following 600 *s* of exposure to apical glucose. It is seen that for apical glucose concentrations up to around 25 mM, the flux through SGLT1 is higher than GLUT2 flux but after that it starts to saturate, while the GLUT2 flux continues to increase and get higher than SGLT1 flux. This behavior looks similar to the previous experimental study Kellett and Helliwell (2000) which at the apical glucose concentration of 50 mM the glucose flux through GLUT2 is about 2 times higher than flux via SGLT1, Thus, varying the level of SGLT1 in the absence of apical GLUT2 is unable to capture the shape and magnitude of the experimental measurements since transport through SGLT1 saturates at an apical glucose concentration of about 25 mM. This suggests that apical GLUT2 is essential to account for the data from Zheng et al. (2012).

### 3.5. Glucose Uptake in Diabetes

**Figure 8:**
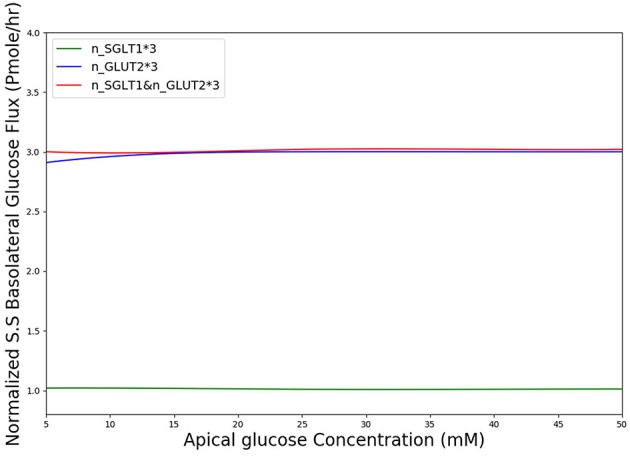
Normalized steady state basolateral glucose flux vs. different stimulus of glucose in the lumen when number of SGLT1 is 3-fold higher(green), number of GLUT2 is 3-fold higher(blue) and number of both SGLT1 & GLUT2 are 3-fold higher(red).

In diabetes, expression levels of SGLT1 and GLUT2 in the small intestine are reported to be increased 3 to 4-fold compared to non-diabetic controls in both human and animal studies (Fedorak et al., 1991; Burant et al., 1994; Dyer et al., 1997, 2002). The surface area of the villi has also been reported to increase in diabetes (Schedl and Wilson, 1971). Together these factors are expected to lead to higher rates of glucose absorption to the blood. However, the magnitude of the effect is not known. We used our developed model to study the effect of a 3-fold elevated SGLT1 and GLUT2 expression levels on glucose flux into the basolateral compartment. Figure 8 shows the ratio of steady state glucose flux into the basolateral compartment, normalized to the flux at baseline conditions over a range of apical glucose concentrations. The increase in glucose absorption is less than the increase in transporter expression levels. For apical glucose concentrations up to 50 mM, 3-fold increase in SGLT1 levels causes a small increase in the basolateral flux over the whole range of apical glucose concentration. This increase is < 1.1 times the baseline value. On the other hand increasing the level of GLUT2 by 3-fold increases the basolateral flux to almost 3 times the baseline value. This increase is observed over the whole range of glucose concentration. The result shows that higher levels of GLUT2 in diabetics may lead to a proportional increase in glucose absorption. In contrast, increases in SGLT1 cause a much smaller increase in absorption. However, SGLT1 may indirectly increase absorption rates since studies have shown that apical GLUT2 expression is dependent on SGLT1 activity (Kellett and Helliwell, 2000).

## 4. Discussion

We have developed a computational model of glucose transport in the enterocyte that includes the full set of relevant transporters. The model is able to reproduce measurements reported in the literature and can be used to answer physiologically relevant questions about glucose uptake rates and mechanisms. In addition, the capabilities of the CellML framework were exploited to compose existing validated models of individual transporters to create the final model, which provides greater confidence in the implementation and facilitates model reuse and sharing.

### 4.1. Comparison With Existing Models

Our model differs from the Thorsen et al. (2014) model in some important respects.

**Figure 9:**
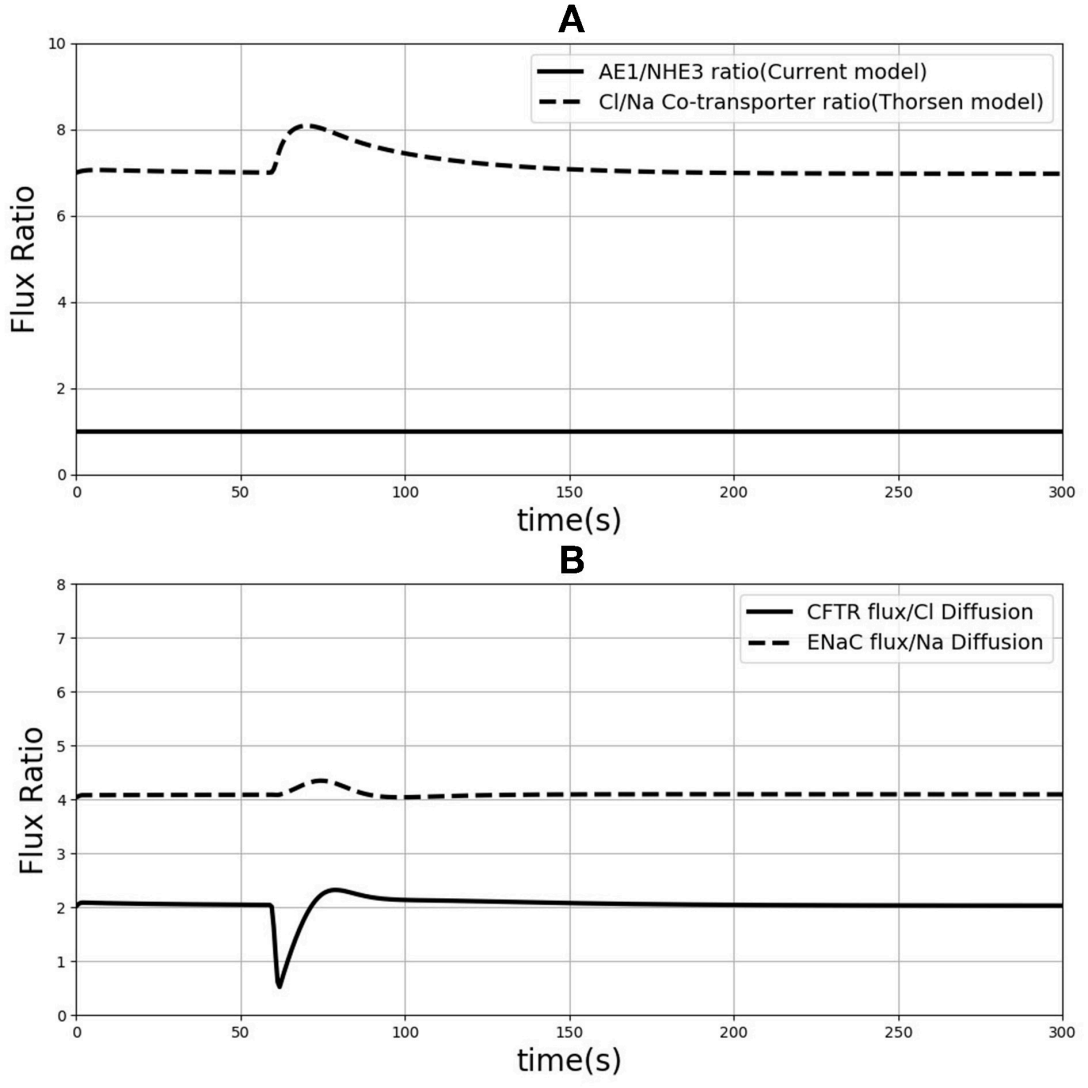
**(A)** Ratio of sodium flux and Chloride flux through NHE3 and AE1 compare to NaCl co-transporter flux in the Thorsen model. **(B)** Chloride flux through CFTR and chloride diffusion ratio compare to the ration of sodium flux through ENaC and sodium diffusion.

One of the differences between the two models is in the treatment of sodium and chloride transport at the apical membrane. Thorsen et al. postulate electro neutral one-for-one fluxes of these ions to account for the sodium-hydrogen (NHE3) and chloride-bicarbonate (AE1) exchangers and use Goldman-Hodgkin-Katz (GHK) diffusion to model ENaC and CFTR. In contrast, our model takes a more general approach by incorporating the individual transport pathways at the apical membrane (Figure 1). We examined the implications of these modeling choices in Figure 9. Figure 9A shows the ratio of the AE1 flux to NHE3 flux for the simulation conditions of Figure 4. In the Thorsen model this ratio is equal to 1, whereas the ratio lies in the range 7–8 in our model. Our decision to explicitly model AE1 and NHE3 offers some advantages and testable consequences. First, our model produces the intracellular pH as an output since H+ concentration is a variable in the model and this provides an additional consistency check. Second, our model can be used to investigate conditions in which the expression/function of AE1 and NHE3 are altered, e.g., impaired absorption in NHE3 knockout mice (Schultheis et al., 1998), reduced chloride absorption and pH imbalance in AE1 mutations (Noonan et al., 2005).

Thorsen et al. used sodium and chloride diffusion through both apical and basolateral membrane of the cell. We replaced them with ENaC and CFTR transporters for sodium and chloride flux in the apical membrane, respectively. Figure 9B shows the ratio of sodium and chloride flux through transporters in our model to the sodium and chloride flux through diffusion in the Thorsen model. It is seen that Chloride flux via CFTR is around 4 times higher than Cl^−^ diffusion and also sodium via ENaC has around 2 times higher flux compared to Na^+^ diffusion in Thorsen model. Thus, the contributions of individual transport pathways are significantly different between the models while still providing similar steady state predictions (Figure 4). By incorporating individual transporters our model offers the flexibility to study effects of drugs or diseases that influence the function of these transporters.

### 4.2. Parameter Choice and Data Fitting

Published values from the literature were used for the majority of transport protein kinetic parameters in our model, with a great deal of information for ohmic models provided by Fong et al. (2016). However, we used a different number of transporters in order to obtain a better fit to the experimental data. These values are shown in the Supplementary Material. The total cellular protein density (*a*) was used as a fitting parameter to match the model predictions of intracellular glucose with the data of Zheng et al. (2012). For both cell types, the fitted value increased with the duration of glucose exposure (Table 4). Since *c*_*pred*_ = *ac*_*expt*_, this indicates that measured uptake increased at a slower rate with exposure time than predicted by the model. Possible reasons could include desensitization or inactivation of transporters and variations in cell protein density between different experiments. Also, the fitted protein densities are lower than indicative values for the mammalian cells (0.1–0.2 g/ml, Milo, 2013). The actual values of *a* do not hold much significance since they depend on the cell volume, which were not estimated in the experiments and were assigned arbitrary, realistic values (volume = 1400μm^3^) (Buschmann and Manke, 1981; MacLeod et al., 1991; Crowe and Marsh, 1993). Given these caveats, the model produces reasonable fits without the requirement of fine tuning.

It was also necessary to make an assumption about the volume of the basolateral compartment in the comparisons with Zheng et al. (2012) as explained before. Rather than treat this as a fitting parameter, we generated a range of model predictions by varying the parameter from small (0.1 times the cell volume) to large (an infinite compartment) values. The model predictions varied by < 5% at short exposure durations and about 50% at long durations (Figure 5) and bracketed the experimental observations in all cases, except few shortest exposures for Caco2. This once again points to the robustness of the model predictions.

### 4.3. Role of Apical GLUT2 in Glucose Uptake and Effect of Time

**Figure 10:**
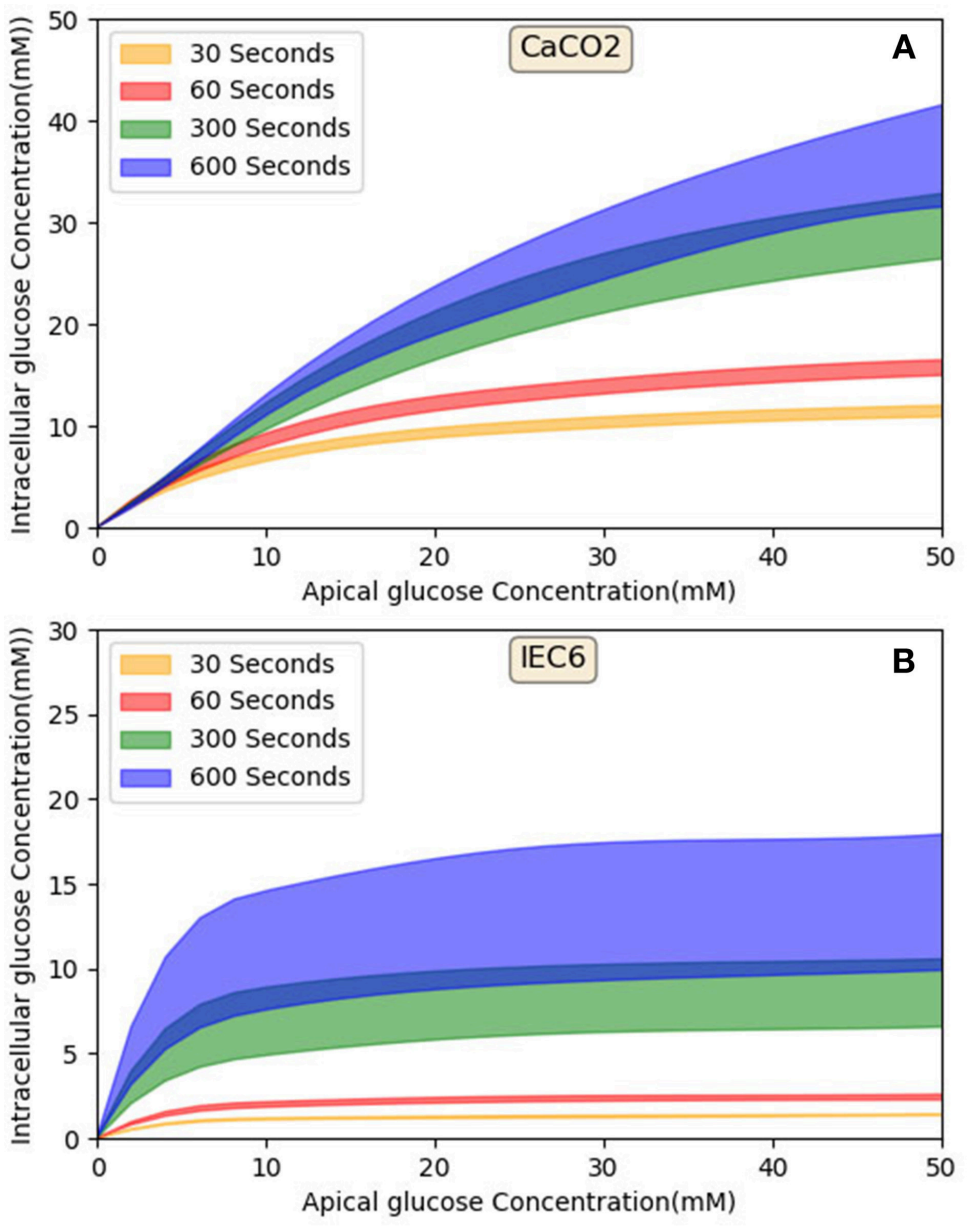
Model output—Intracellular glucose concentration vs. Extracellular glucose concentration in **(A)** Caco2 cell line and **(B)** IEC6 cell line.

Figure 10 shows that in both cell lines at short exposure times the glucose uptake has a tendency to be saturated (in 30 and 60 s), in longer term (>300 s) Caco2 shows non-saturation glucose uptake (Figure 10A) however IEC6 has a greater tendency to level off even at higher apical glucose concentration (Figure 10B). It has been reported that increasing the glucose concentration in the lumen can cause the apical translocation of GLUT2 (Scow et al., 2011) however, in our model results do not require acute translocation of GLUT2 to the apical membrane. Also Western blots experiments showed higher level of GLUT2 expression in higher extracellular glucose concentration (Kellett and Brot-Laroche, 2005); however, in our model density of apical GLUT2 was the same in different concentrations and exposure times. This shows that apical GLUT2 is highly crucial in order to explain the behavior of intracellular glucose absorption.

According to Figure 8, in diabetic patients GLUT2 plays much more important role in the increased glucose absorption compared to SGLT1 regarding the number of transporters. This is in fact a very interesting finding which could be a potential subject of future research into the role of glucose transporter expression levels in diabetic patients.

In summary, we have developed an integrative model of glucose uptake in the enterocyte that incorporates mechanistic descriptions of all relevant transporters and validated it against published measurements and models with minimal parameter tuning. The work utilizes the CellML modeling framework and the Physiome Model Repository to provide a portable, publically available implementation that facilitates sharing, reuse and extension of the model. We expect that the model will provide insight into transport pathways and guide the design and interpretation of experiments to generate and test hypotheses. We have used the model to determine the relative contribution of SGLT1 and GLUT2 to glucose absorption under a range of conditions. We have also evaluated the consequences of altered SGLT1 and GLUT2 expression in diabetes on glucose absorption rates. Potential applications in the future can include predictive modeling of the effect of drugs such as SGLT1 and GLUT2 inhibitors on glucose uptake and ion transport. This model of cellular uptake can be coupled with models of blood flow and metabolism to develop a more complete predictive framework of glucose homeostasis in the body (Nickerson et al., 2015).

## Author Contributions

NA, SS, DPN, PJH, and VS contributed conception and design of the study. NA performed the statistical analysis and modeling, wrote the first draft of the manuscript along with sections of the manuscript. VS and SS checked the model and validation. All authors contributed to manuscript revision, read and approved the submitted version.

### Conflict of Interest Statement

The authors declare that the research was conducted in the absence of any commercial or financial relationships that could be construed as a potential conflict of interest.
