# Supplementary material for: Computational Modeling of Glucose Uptake in the Enterocyte
Source: Front Physiol. 2019 Apr 12;10:380. doi: 10.3389/fphys.2019.00380 (PMC6473069; doi:10.3389/fphys.2019.00380)
Supplement: Supplementary file 1 [file Data_Sheet_1.PDF]

Supplementary Material: Computational Modelling of Glucose Uptake in the Enterocyte

Nima Afshar [1,*], **Soroush Safaei** [1], **David Nickerson** [1], **Peter Hunter** [1] **and Vinod Suresh** [1,2]
*Correspondence:
Vinod Suresh
v.suresh@auckland.ac.nz

1 MODEL EQUATIONS

Membrane Potential

Based on the conservation of charge, summation of all ionic currents through the apical or basolateral membrane divided by the cell capacitance gives the membrane potential. It includes all the currents via electrogenic ion channels and paracellular pathway. Electroneutral transporters do not play any role in the net flux of charge. A positive ion entering the cell at either the apical or basolateral membrane is treated as a positive current

$$\frac{dv_{mc}}{dt} = \frac{(I_{Na_{SGLT1}} + I_{BK} + I_{CFTR} + I_{D_{P_{Na}}} + I_{D_{P_K}} + I_{D_{P_{Cl}}})}{Capacitance} \tag{S1}$$

$$\frac{dv_{sc}}{dt} = \frac{(I_{KCNQ} + I_{CLC2} + I_{K_{NaK}} - I_{Na_{NaK}} - I_{D_{P_{Na}}} - I_{D_{P_K}} - I_{D_{P_{Cl}}})}{Capacitance} \tag{S2}$$

Ionic balance equation

Intracellular ion concentrations are calculated based on the summation of all the inlet and outlet fluxes divided by the cell volume. The flux J_m of species m is equal to the current I_m of that species divided by the Faraday number. The equation for pH uses the buffering capacity β (mM/pH unit). Expressions for the individual currents and fluxes are found the in the references cited in Table 1.

$$\frac{dNa_i}{dt} = (\frac{1}{v_{cell}}).(J_{Na_{SGLT1}} - J_{Na_{NaK}} + J_{ENaC} + J_{NaKCC} + J_{Na_{NBC}} + J_{Na_{NHE3}}) \tag{S3}$$

$$\frac{dglucose_i}{dt} = (\frac{1}{v_{cell}}).(J_{A_{GLUT2}} + J_{Gl_{SGLT1}} - J_{B_{GLUT2}}) \tag{S4}$$

$$\frac{dK_i}{dt} = (\frac{1}{v_{cell}}).(J_{K_{NaK}} + J_{NaKCC} + J_{IK} + J_{BK}) \tag{S5}$$

$$\frac{dCl_i}{dt} = (\frac{1}{v_{cell}}).(J_{NaKCC} + J_{Cl_{AE1}} - J_{CFTR} - J_{CLC2}) \tag{S6}$$

$$\frac{dpH_i}{dt} = (\frac{1}{v_{cell}\beta}).(J_{HCO3_{NBC}} - J_{H_{NHE3}} - J_{HCO3_{AE1}}) \tag{S7}$$
